# Human Leptospirosis Trends, the Netherlands, 1925–2008

**DOI:** 10.3201/eid1903.111260

**Published:** 2013-03

**Authors:** Marga G.A. Goris, Kimberly R. Boer, Tamara A.T.E. Duarte, Suzanne J. Kliffen, Rudy A. Hartskeerl

**Affiliations:** Author affiliation: Royal Tropical Institute (KIT), Amsterdam, the Netherlands

**Keywords:** human leptospirosis, Leptospira spp., bacteria, epidemiology, epidemiologic trends, diagnosis, travelers, passive serologic surveillance, sex, accidents, recreational activities, zoonoses, the Netherlands

## Abstract

To increase knowledge of leptospirosis in the Netherlands and identify changing trends of this disease over time, we analyzed historical passive surveillance reports for an 84-year period (1925–2008). We found that 2,553 mainly severe leptospirosis cases were diagnosed (average annual incidence rate 0.25 cases/100,000 population). The overall case-fatality rate for patients with reported leptospirosis was 6.5% but decreased over the period, probably because of improved treatment. Ninety percent of reported leptospirosis cases were in male patients. Most autochthonous leptospirosis infections were associated with recreational exposures, but 15.5% of the cases were attributed to accidents that resulted in injury and to concomitant water contact. Since the end of the 1950s, the proportion of imported infections gradually increased, reaching 53.1% of the total during 2005–2008. Most (80.1%) imported infections were associated with sporting and adventurous vacation activities.

Leptospirosis is a zoonotic disease caused by infection with *Leptospira* spp. bacteria (*1*). Pathogenic leptospires live in the kidneys of many mammalian hosts, including rodents, insectivores, and livestock. Leptospires are shed into the environment, where they can survive for several months in favorable (warm and wet) conditions. Thus, leptospirosis is particularly endemic to warm and humid tropical and subtropical regions (*2*). Humans are infected by direct contact with infected animals or indirectly by contact with a contaminated environment.

Leptospirosis is an emerging public health problem globally (*3**–**6*). However, this disease is often overlooked because it is difficult to clinically diagnose and because and laboratory-based diagnosis is cumbersome. Because mild leptospirosis frequently goes unrecognized and notification systems are mostly absent, the global incidence of leptospirosis is underestimated. An international survey conducted by the International Leptospirosis Society reported ≥350,000 cases of severe leptospirosis annually (*7*). This estimate is supported by data from an assessment of the global incidence of leptospirosis (*8*), which indicated a mean global incidence rate for leptospirosis of 5 cases/100,000 population.

In Europe, leptospirosis has been studied and diagnosed since the 1920s. Historical reviews from Germany (*9*) and France (*10*) have contributed to a better understanding of the epidemiology of leptospirosis. In the Netherlands, passive surveillance of human leptospirosis began in 1924. Reporting of cases of this disease is mandatory, and laboratory diagnosis has been centralized in 1 institution. To increase knowledge of leptospirosis, we analyzed historical passive surveillance reports in the Netherlands for 84 years (1925–2008) to determine changing trends of this disease over time.

## Passive Surveillance

The Royal Tropical Institute (KIT) in Amsterdam is associated with the World Health Organization/Food and Agricultural Organization/World Organisation for Animal Health and the National Collaborating Centre for Reference and Research on Leptospirosis (NRL), which confirms ≈99% of the suspected cases of leptospirosis in the Netherlands. Detailed records on serologic, clinical, and epidemiologic features are archived at the NRL. Since 1928, leptospirosis has been a mandatory reportable disease in the Netherlands (*11*). A case of leptospirosis is defined as laboratory confirmation of infection as described in this report and by Hartskeerl (*12*) and fever or 2 of the following signs and symptoms: shivering, headache, muscle pain, conjunctival injection, bleeding in skin and mucosa, rash, jaundice, myocarditis, meningitis, renal failure, pulmonary hemorrhage with respiratory failure.

## Study Population

Reportable disease data for leptospirosis are compiled from passive surveillance reports received for the entire population of the Netherlands. General practitioners and consulting clinicians suspecting leptospirosis send clinical specimens to the NRL for laboratory evaluation. During the period covered by this review, the total number of samples submitted for testing was estimated to be ≈50,000.

## Changes in Laboratory Diagnosis

Laboratory tests have changed over time. However, all diagnoses in the Netherlands have been based on identification of leptospires by culture or antibodies against *Leptospira* spp. by agglutination tests (*12*). During 1924–1963, culture of patient specimens was routinely performed by inoculation of blood or urine into guinea pigs or hamsters. In 1964, in vitro culturing was introduced and has been used exclusively since 1972.

Beginning in 1924, the agglutination test was used for diagnosis of leptospirosis; the test was performed as described by Martin and Pettit (*13*) using serovars Pyrogenes and Rachmat and unidentified isolates from patients. Serovar Copenhageni was included in 1927, serovars Icterohaemorrhagiae and Canicola in 1934, serovar Grippotyphosa in 1941, serovars Pomona and Bataviae in 1942, and serovar Ballum in 1945. During 1960–1990, the panel was increased by the addition of serovars Tarassovi (1961), Poi (1963), Bratislava (1964), Saxkoebing (1964), Hardjo-prajitno (1980), Hebdomadis (1981), Hardjo-bovis (1983), Proechimys (1987), and Sejroe (1987). This panel was later supplemented with serovars Ballico, Celledoni, Cynopteri, Mini, Panama, and Shermani to include the representative serovars that that cause leptospirosis worldwide (*1*).

The agglutination test was modified in 1954 in accordance with recommendations of Wolff (*14*) and in 1978 in accordance with recommendations of Cole et al. (*15*). An in-house IgM ELISA was introduced in 1984 (*16*). Laboratory diagnosis is currently based on a positive culture, a microscopic agglutination test titer ≥160 and IgM ELISA titer ≥80, or seroconversion (*12*). Most presumptive infecting serogroups are deduced from the highest titers against ≥1 serovars in the microscopic agglutination test. Such titers are only indicative for serogroups (*17*). Therefore, we report data on infecting serogroups. In cases in which a leptospiral isolate has been typed to serovar level, the corresponding serogroup has been used.

We also assessed differences between autochthonous and imported leptospirosis infections. Autochthonous infections are those most likely acquired in the Netherlands. Imported infections are those most likely acquired during a visit to another country <1 month before the day of symptom onset.

## Data Collection

During 1924–1964, when a case of leptospirosis was confirmed in the Netherlands, demographic, epidemiologic, and clinical data were collected by using a standardized questionnaire sent to the consulting physician by the NRL. When persons with confirmed leptospirosis were hospitalized, the physician was requested to send a copy of the patient’s discharge letter to the NRL. Because of changes in privacy laws, during 1964–1999 questionnaires were sent directly to the patients, but since 1999, questionnaires and informed consent forms have been sent to consulting physicians.

## Data Analysis

Archived information was entered into a database by using SPSS version 15.0 software (IBM, Armonk, NY, USA). Data were analyzed for trends over time, differences between autochthonous and imported infections, and differences related to sex of the patients.

Total annual incidence for male and female patients was calculated by using the population of the Netherlands as obtained from the Dutch Central Bureau for Statistics (The Hague, the Netherlands). Annual case-fatality rates (CFRs) were calculated by dividing the number of deaths by the total number of confirmed cases per year. Patient data on hospitalization, antimicrobial drug treatment (available since 1950), hemodialysis (available since 1961), and treatment in an intensive care unit (ICU) (available since 1955) were used to give an overview of clinical management of leptospirosis in the Netherlands. Missing data were recoded as “no” for the analysis.

To assess the severity of disease, data were analyzed for changes over time for 10-year intervals during 1925–2008 by using the χ^2^ test for trend. Severe leptospirosis was defined as disease for which hospitalization, admission to an ICU, or dialysis was indicated, or for which death occurred. Severity calculations are limited to the period when such supportive treatment was available. These characteristics were analyzed by using the χ^2^ test, Student *t* test, or Fisher exact test when appropriate.

Differences between autochthonous and imported infections regarding patient characteristics, likely source of exposure, treatment course, and infecting serogroups were tested by using the χ^2^ test and Fisher exact test for values <5. Likely source of exposure was determined by type of contact (recreational activities, accidental, job-related) and route of infection (water, animal or other source) source and route are mutually exclusive, in contrast to assessment of animal hosts.

We examined whether bacterial exposure and disease severity differed by patient sex. Patient data concerning mean age, imported disease, type of contact, and treatment course are presented by sex of the patient. Differences between male and female patients were analyzed by using the χ^2^ test. A p value ≤0.05 was considered significant.

## Ethical Issues

This study was exempted from ethical review of human subject research by the Medical Ethical Review Committee of the Academic Medical Centre, University of Amsterdam (protocol W12_075#12.17.0092). All data have been de-identified and were not attributable to individual patients.

## Cases

During 1925–2008, the NRL reported 2,588 leptospirosis infections. Thirty-five case-patients were excluded: 22 living outside the Netherlands, 1 whose case was reported more than once, and 12 whose cases were retrospectively reclassified. Thus, the study sample comprised 2,553 confirmed case-patients. An overview of patient characteristics by autochthonous and imported infections is shown in Tables 1 and 2. Male patients accounted for 91.1% of all infections. Deaths were almost exclusively reported in men who had autochthonous infections with serogroup Icterohaemorrhagiae. Most (80.8%) imported leptospirosis cases and a substantial (44.4%) proportion of autochthonous cases were associated with recreational activities. A substantial number of infections (14.4%) were attributed to injury (i.e., traffic accidents and concomitant water exposure).

**Table 1:** Characteristics for case-patients with leptospirosis, the Netherlands, 1925–2008*

Characteristic	Total cases, n = 2,553	Autochthonous cases, n = 2,231†	Imported cases, n = 318†	p value
Male sex‡	2,306 (91.1)	2,025 (91.6)	278 (87.4)	0.014
Mean (SD) age, y‡	33.8 (17.1)	34.0 (17.5)	32.6 (14.4)	0.848
Type of contact				
Recreational activity	1,250 (49.0)	990 (44.4)	257 (80.8)	<0.001
Job-related	685 (26.8)	664 (29.8)	21 (6.6)	<0.001
Accident	367 (14.4)	345 (15.5)	22 (6.9)	<0.001
Unknown	250 (9.8)	232 (10.4)	18 (5.7)	
Likely route of infection				
Water	1,457 (57.1)	1,219 (54.6)	236 (74.2)	<0.001
Water and animals	500 (19.6)	446 (20.0)	53 (16.7)	0.162
Animals	351 (13.7)	346 (15.5)	4 (1.3)	<0.001
Other§	16 (0.6)	14 (0.6)	2 (0.6)	1.000
Unknown	229 (9.0)	206 (9.2)	23 (7.2)	
Host exposure¶	(n = 851)	(n = 792)	(n = 57)	
Rats	443 (52.1)	411 (51.9)	32 (56.1)	<0.535
Mice	123 (14.6)	120 (15.2)	3 (5.3)	0.004
Other rodents	31 (3.6)	28 (3.5)	3 (5.3)	0.789
Cows	231 (27.1)	224 (28.3)	6 (10.5)	<0.001
Dogs	172 (20.2)	158 (19.9)	13 (22.8)	0.603
Other animals	185 (21.7)	168 (21.2)	16 (28.1)	0.088
Serogroup				
Icterohaemorrhagiae	1,702 (66.7)	1,588 (71.2)	111 (34.9)	<0.001
Grippotyphosa	196 (7.7)	174 (7.8)	22 (6.9)	0.595
Sejroe	128 (5.0)	116 (5.2)	12 (3.8)	0.771
Canicola	93 (3.6)	87 (3.9)	6 (1.9)	0.230
Pomona	54 (2.1)	45 (2.0)	9 (2.8)	0.107
Autumnalis	16 (0.6)	1 (0.1)	15 (4.7)	<0.001
Bataviae	11 (0.4)	0	11 (3.5)	<0.001
Other#	54 (2.1)	12 (0.5)	42 (13.2)	<0.001
Unknown	299 (11.7)	208 (9.3)	90 (28.3)	<0.001
Cultures performed	1,335 (52.3)	1,151 (51.6)	182 (57.2)	0.060
Positive result	306 (22.9)	256 (22.2)	49 (26.9)	0.162

*Values are no. (%) unless otherwise indicated. †Data for autochthonous and imported cases could not be obtained for 4 patients.  ‡Sex was recorded for 2,532 patients: 2,210 patients with autochthonous cases and 318 patients with imported cases. Age was recorded 2,427 patients; 2,105 patients with autochthonous cases and 381 patients with imported cases. §Laboratory accidents or contact with mud/soil. ¶Animal specified is not mutually exclusive; 1 patient could have been in contact with >1 animal. #For total infections, Australis (n = 12), Celledoni (n = 7), Sejroe/Hebdomadis/Mini complex (n = 7), Javanica (n = 6), Pyrogenes (n = 6), Hebdomadis (n = 4), Shermani (n = 4), Ballum (n = 2), Cynopteri (n = 2), Tarassovi (n = 2), Celledoni/Javanica complex (n = 1), Mini (n = 1). For autochthonous infections, Australis (n = 6), Ballum (n = 2), Tarassovi (n = 2), Javanica (n = 1), Sejroe/Hebdomadis/Mini complex (n = 1). For imported infections, Celledoni (n = 7), Australis (n = 6), Pyrogenes (n = 6), Sejroe/Hebdomadis/Mini complex (n = 6), Javanica (n = 5), Hebdomadis (n = 4), Shermani (n = 4), Cynopteri (n = 2), Celledoni/Javanica complex (n = 1), Mini (n = 1).

**Table 2:** Treatment parameters for and deaths among case-patients with leptospirosis, the Netherlands, 1925–2008*

Characteristic	No. (%) total cases	No. (%) autochthonous cases	No. (%) imported cases	No. patients	p value
Deaths					
Total	166 (6.5)	162 (7.3)	4 (1.3)	2,553	<0.001
Male patients	160 (97.0)	156 (96.9)	4 (100.0)	2,306	0.861
Treatment course†					
Hospitalization	1,851 (72.5)	1,612 (72.3)	235 (73.9)	2,553	0.539
Antimicrobial drugs	1,216 (68.5)	994 (68.2)	219 (69.5)	1,597‡	0.652
Dialysis	119 (8.8)	103 (9.8)	15 (5.0)	796§	0.009
ICU	106 (6.5)	83 (6.3)	23 (7.3)	626¶	0.485

*ICU, intensive care unit.  †Percentages were calculated from available data (all missing values were regarded as no). It was assumed that analyzing data that contained large amounts of missing values would result in findings that could not be generalized and lead to substantial overestimated values. ‡Antimicrobial drug data were available for 1,597 of 1,776 infected patients, 1,306 of 1,457 patients with autochthonous cases, and 288 of 315 patients with imported cases (January 1950–December 2008). Before 1950, treatment with antimicrobial drugs was not expected to be used. §Dialysis treatment data were available for 796 of 1,360 infected patients, 559 of 1,053 patients with autochthonous cases, and 234 of 303 patients with imported cases (January 1961–December 2008). Before 1961, dialysis treatment was not expected to be used. ¶ICU data were available for 626 of 1,641 infected patients, 441 of 1,324 patients with autochthonous cases, and 183 of 313 patients with imported cases (April 1955–December 2008). Before 1955, treatment in an ICU was not expected to be used.

## Trends over Time

The average annual incidence rate of leptospirosis in the Netherlands was 0.25 cases/100,000 population (Figure). For male patients, the average incidence was 0.46 cases/100,000 boys and men, which is >10-fold higher than the rate for female patients (0.04 cases/100,000 girls and women).

**Figure:**
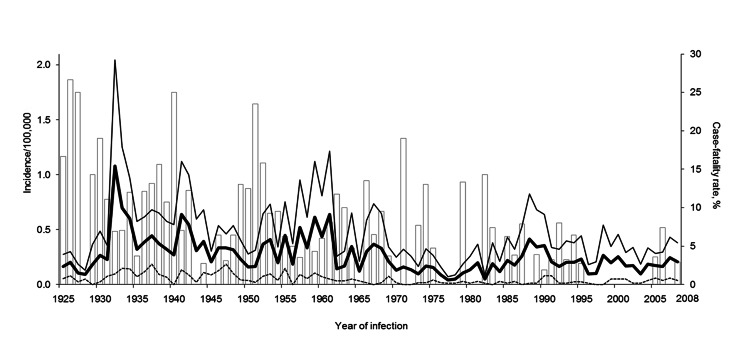
Incidence rates of leptospirosis, the Netherlands, 1925–2008. White bars indicate case-fatality rate (percentage of deaths/no. of confirmed cases), thick black line indicates total incidence rate (no. cases/100,000 population), thin black line indicates incidence rate among male patients (no. cases in male patients/100,000 male population), and dashed line indicates incidence rate among female patients (no. cases in female patients/100,000 female population). The total population of the Netherlands was 7.3 million in 1925, 8.4 million in 1935, 9.2 million in 1945, 10.7 million in 1955, 12.2 million in 1965, 13.6 million in 1975, 14.5 million in 1985, 15.4 million in 1995, and 16.3 million in 2005.

The mean (SD) age of patients was 33.8 (17.1) years. There was a gradual increase in the mean (SD) age over time: 29.1 (14.6) years during 1925–1934 to 38.0 (16.1) years during 2005–2008 (p<0.001).

Although leptospirosis is endemic to most of the Netherlands, there have not been any large outbreaks. Peak periods of incidence rate increases were seen in 1932 (1.08 cases/100,000 population), 1941 (0.64 cases/100,000 population), and 1961 (0.64 cases/100,000 population), and to a lesser extent in 1967 (0.37 cases/100,000 population) and 1988 (0.42 cases/100,000 population) (Figure).

During 1925–2008, a total of 166 persons with leptospirosis died (CFR 6.5%) (Table 2). The annual number of deaths decreased over time, probably as a result of the introduction of dialysis treatment in 1961; there were 67 deaths (10.5%) in the first 20 years and 21 (3.9%) during 1975–1994 (p<0.001). Since 1995, the NRL has only recorded 5 (1.2%) deaths. During 1925–2008, the mean (SD) age of patients who died was 49.2 (15.6) years, and the mean (SD) age of patients who survived was 32.8 (16.9) years (p<0.001). The mean (SD) age of patients who died increased from 43.6 (16.0) years during 1925–1934 to 64.2 (12.7) years over the last 4 years of the study (p<0.05).

A total of 1,851 (72.5%) of the 2,553 patients were hospitalized. The proportion of patients hospitalized increased from 37.4% during 1925–1934 to 92.1% during 1955–1964 (p<0.001) and then decreased to 74.4% during 2005–2008 (p<0.001). Overall, 6.5% of patients were treated in an ICU and 8.8% received dialysis treatment.

The first ICU admissions for leptospirosis were reported in 1978. During 1975–1984, a total of 7.5% of patients were reported as being admitted to an ICU. The percentage of ICU admissions increased to 17.8% during 2005–2008. Dialysis treatment was introduced and recorded for the first time in 1961; its use increased markedly from 3 (2.0%) patients with leptospirosis during 1955–1964 to 16 (12.4%) patients during 2005–2008. Widespread use of penicillin to treat leptospirosis first began during World War II. Treatment with antimicrobial drugs increased from 53.3% of patients during 1945–1954 to 83.7% of patients during 2005–2008.

During 1925–2008, the main infecting serogroup in autochthonous cases was Icterohaemorrhagiae (1,588 identifications; 71.2%). Other common serogroups were Grippotyphosa (174 identifications; 7.8%), Sejroe (116 identifications; 5.2%), Canicola (87 identifications; 3.9%), and Pomona (45 identifications; 2.0%). All infections with serogroup Canicola occurred during the first 50 years of the study, and there was a peak during 1945–1954. Serogroup Icterohaemorrhagiae appeared to be the major cause of fatal leptospirosis, followed by serogroup Canicola (Table 3). Within the group of patients infected with serogroup Icterohaemorrhagiae, 90 patients died (CFR 5.3%). This number represents 94.7% of the patients who died for whom the infecting serogroup is known. 

**Table 3:** Treatment course and deaths, by infecting *Leptospira* spp. serogroups, for case-patients with leptospirosis, the Netherlands, 1925–2008*

Serogroup	No. patients	No. (%) hospitalized	No. (%) treated with dialysis	No. (%) treated in ICU	No (%) died
Icterohaemorrhagiae	1,702	1,311 (77.0)	90 (11.9)	68 (7.0)	90 (5.3)
Grippotyphosa	196	124 (63.3)	3 (2.2)	2 (1.2)	0
Sejroe	128	48 (37.5)	3 (2.4)	2 (1.6)	1 (0.8)
Canicola	93	60 (64.5)	0	0 (0.0)	3 (3.2)
Pomona	54	40 (74.1)	3 (5.6)	7 (13.0)	0
Other	81	55 (67.9)	3 (3.8)	6 (7.6)	1 (1.9)
Unknown	299	213 (71.2)	17 (8.6)	21 (9.3)	71 (23.7)

*ICU, intensive care unit. Data were available for 2,553 patients who were hospitalized or died, 1,360 patients treated with dialysis, and 1,641 patients treated in an ICU.

The CFR for patients infected with serogroup Canicola was 3.2%. None of the patients infected with serogroup Pomona died, although the percentages of hospitalization, ICU admission, and dialysis treatment were higher among these patients. Because all patients infected with serogroup Canicola were observed before 1967, dialysis and ICU treatment were not available for these patients, but absence of these treatments does not indicate milder clinical illness. Serogroups Grippotyphosa and Sejroe appeared to cause less severe disease, although 1 patient infected with serogroup Sejroe died (Table 3). Fatal leptospirosis can have a rapid, fulminate course, which often makes identification of infecting serogroups impossible. Information for the causative serogroup was available for only 95 of the 166 patients who died. The average CFR was 4.2% for patients in whom the infecting serogroup was determined and 23.7% for patients in whom the infecting serogroup was not identified.

## Autochthonous versus Imported Leptospirosis

In the Netherlands, the total number of leptospirosis patients infected outside the country through 2008 was 318 (12.5% of all reported patients). The annual proportion of imported leptospirosis cases has gradually increased over time; >50% of all infections during 2005–2008 were acquired outside the Netherlands (Table 4). In the early years of the study, a substantial proportion of imported infections occurred after exposure in other countries in Europe, mainly during vacations. Since the mid-1970s, the number of leptospirosis infections acquired outside Europe has increased markedly, mostly from exposures in Asia (134 cases; 42.1%), notably Thailand (Table 4). More than 80% of the imported leptospirosis infections were associated with water-related sport and adventure activities, such as white-water rafting. In contrast, 44.4% of the autochthonous infections were attributed to recreational activities (Table 1), 29.8% were attributed to occupational exposures, and 15.5% were attributed to accidents. Except during 1985–1994, the ratio of infections related to recreational activities, occupations, and accidents has remained similar over time.

**Table 4:** Characteristics for case-patients with leptospirosis, the Netherlands and other regions, 1925–2008*

Characteristic	Period								
	1925–1934	1935–1944	1945–1954	1955–1964	1965–1974	1975–1984	1985–1994	1995–2004	2005–2008
No. case-patients	289	352	271	434	259	159	379	281	129
Hospitalized, %	37.4	68.8	78.6	92.1	83.8	84.3	62.5	72.6	74.4
Antimicrobial drug treatment, %	NA	NA	53.3	63.8	61.8	57.9	72.3	82.9	83.7
Dialysis, %	NA	NA	0	2.0	5.0	17.6	8.2	10.0	12.4
ICU, %	NA	NA	0	0.0	0.0	7.5	6.6	16.4	17.8
CFR, %	11.1	9.9	9.6	6.0	8.1	3.8	4.0	0.4	3.1
Imported infections	0	2 (0.6)	3 (1.1)	20 (4.6)	22 (8.5)	17 (0.7)	92 (24.3)	94 (33.5)	68 (53.1)
Europe	0	2	2	19	20	9	40	27	13
Asia	0	0	1	0	1	4	38	48	42
Sub-Saharan Africa	0	0	0	0	0	0	2	4	1
South America	0	0	0	0	1	4	6	7	7
Central and North America	0	0	0	0	0	0	2	6	5
Middle East	0	0	0	1	0	0	0	1	0
Australia	0	0	0	0	0	0	0	1	0
Likely route of infection									
Water									
Autochthonous	222 (92.8)	251 (82.3)	194 (78.7)	333 (87.4)	189 (88.3)	111 (79.9)	157 (60.4)	162 (88.5)	46 (79.3)
Imported	NA	2 (100)	2 (66.7)	18 (94.7)	19 (95.0)	15 (93.8)	84 (98.8)	89 (100.0)	60 (98.4)
Animal									
Autochthonous	15 (6.3)	52 (17.0)	48 (19.5)	44 (11.5)	25 (11.7)	27 (19.4)	103 (39.6)	21 (11.5)	11 (19.0)
Imported	NA	0	0	1 (5.3)	1 (5.0)	1 (6.2)	1 (1.2)	0	0
Other									
Autochthonous	2 (0.8)	2 (0.7)	4 (1.6)	4 (1.0)	0	1 (0.7)	0	0	1 (1.7)
Imported	NA	NA	1 (33.3)	0	0	0	0	0	1 (1.6)
*Values are no. (%) except as indicated. ICU, intensive care unit; CFR, case-fatality rate; NA, not available.									

## Sex Differences

Of 2,532 patients, 2,306 (91.1%) were male patients and 226 (8.9%) were female patients (Table 5). On the basis of CFR data, male patients were more likely to have had a more severe leptospirosis infection; 160 male patients died (CFR 6.9%) and 6 female patients died (CFR 2.7%) (Table 2). In addition, a higher percentage of male patients were treated with dialysis (Table 5). The greater severity of leptospirosis among male patients does not appear to be attributable to infections caused by more virulent serovars: 67.2% of male patients were infected with serogroup Icterohaemorrhagiae compared with 61.1% of female patients (p = 0.06). However, male patients were older than female patients: mean (SD) was 34.5 (17.0) years for male patients and 26.4 (16.5) years for female patients (p<0.001).

**Table 5:** Characteristics for 2,532 case-patients with leptospirosis, by sex, the Netherlands, 1925–2008*

Characteristic	Male patients, n = 2,306	Female patients, n = 226	p value
Mean age, y (SD)	34.5 (17.0)	26.4 (16.5)	<0.001
Patients with imported cases	278 (12.1)	40 (17.8)	0.014
Type of contact			
Recreational activity	1,086 (47.1)	162 (71.7)	0.001
Job-related	666 (28.9)	18 (8.0)	<0.001
Accident	339 (14.7)	27 (11.9)	0.235
Unknown	215 (9.3)	19 (8.4)	
Treatment course†			
Hospitalization	1,683 (73.0)	163 (72.1)	0.781
Dialysis‡	118 (9.3)	1 (1.1)	0.007
ICU§	102 (6.7)	4 (3.4)	0.063
Death†	160 (6.9)	6 (2.6)	0.013

*Values are no. (%) unless otherwise indicated. Data for age and sex were available for 2,422 patients, and data for autochthonous or imported infections and sex were available for 2,528 patients. ICU, intensive care unit. †Percentages were calculated from available data (missing values were regarded as no). It was assumed that analyzing data that contained large amounts of missing values would result in findings that could not be generalized and lead to substantial overestimated values. ‡Dialysis data were available for 795 of 1,360 patients (732 of 1,263 male patients and 63 of 93 female patients) (January 1961–December 2008). Before 1961, dialysis was not expected to be used. §ICU data were available for 625 of 1,641 patients (573 of 1,518 male patients and 52 of 119 female patients) (April 1955–December 2008). Before 1955, ICU was not expected to be used.

## Conclusions

Leptospirosis is endemic to the Netherlands. During the study period, the average incidence was 0.25 cases/100,000 population. The reported incidence probably reflects the more severe end of the clinical spectrum for leptospirosis because mild forms of this disease are more likely to go unrecognized (*1**,**8**,**18**,**19*). In the 84 years covered by this study, there were 5 years with notably increased annual incidences. The peaks in 1932 and 1941 coincided with the global economic depression and compulsory reporting of leptospirosis (1932) (*20*) and with World War II (1941). The increase in 1988 was associated with a dairy fever outbreak among farmers who were infected while handling leptospirosis-infected cattle. (*18**,**21*). No specific situations or events are known to be associated with the peaks in 1961 and 1968.

The overall CFR of 6.5% was high and exceeded CFRs reported in some countries with a higher prevalence of leptospirosis (*3**,**4**,**22**,**23*). The CFR decreased over the analysis period, probably because of improved treatments. No deaths were recorded during 1995–2005. However, this finding was probably caused by underreporting because transient stringent privacy regulations hampered identification of deaths. Thus, the actual average CFR might be higher than calculated for years after 1999.

An explanation for the high CFR might be that most (71.2%) autochthonous leptospirosis cases identified in the Netherlands were caused by more virulent serovars of serogroup Icterohaemorrhagiae; infections with this serogroup are less common among imported cases. Another reason might be that clinicians in the Netherlands are more proactive in treating severe leptospirosis in travelers than in persons with locally acquired infections. Potentially fulminant leptospirosis leading to rapid death, in combination with limited diagnostic potential in countries to which this disease is endemic, often prevents confirmation of leptospirosis cases (*24*) and might lead to underestimation of the CFR (*3**,**4*). Serogroups Grippotyphosa, Sejroe, Canicola, and Pomona frequently cause leptospirosis in the Netherlands. Infections with serovar Hardjobovis in serogroup Sejroe were found mainly in dairy fever cases among farmers from 1985 until early 1990. Infections with serogroup Pomona were more recent and mostly caused by the newly identified serovar Mozdok (*25*).

Infections with serogroup Canicola were not found after 1966. This finding was probably caused by introduction of bivalent Copenhageni/Canicola canine vaccines. Dogs are the reservoir of serovar Canicola, and vaccination interfered with the transmission cycle, resulting in elimination of serovar Canicola infections in the dog population in the Netherlands (*26*). Lack of dialysis and ICU treatments in patients infected with serogroup Canicola was mainly caused by absence of these treatments and does not indicate less severe or underestimated symptoms.

Recreational activities accounted for most (44.4%) autochthonous leptospirosis cases in this analysis, and ≈80% of imported infections were acquired during recreational water contact. Our data indicate that infections acquired during holidays in tropical countries are increasing. An increase in the incidence of leptospirosis related to exposures in tropical countries has been reported (*9*). Therefore, clinicians should consider leptospirosis in the differential diagnosis for patients with a febrile illness and a history of travel abroad.

A total of 15.5% of autochthonous and 6.9% of imported cases were reportedly caused by accidents with water exposure, indicating a need to further study this issue (*22*). In Germany accidental exposure has been reported and represented 3% of all reported cases during 1997–2000 (*9*).

The total incidence of leptospirosis in the Netherlands showed a small decrease over the 84-year study period. A decreasing trend in incidence has also been observed in France by Baranton and Postic (*10*), albeit, more pronounced. These authors attribute the decrease to changes in lifestyle and the rural environment. In the Netherlands, overall incidence is not decreasing as rapidly because of the increase in imported cases. The decrease in autochthonous infections in the Netherlands may reflect the success of the surveillance system and associated dairy control measures and vaccination of dogs, as shown by elimination of infections with serogroup Canicola since 1974 (*12**,**18**,**21*).

During the study period, most leptospirosis cases in the Netherlands were in male patients. However, surveillance systems based on passive reporting are biased toward including more severe cases, which are found more often in male patients (*27*). Therefore, if milder symptoms develop in female patients, these cases might also be more likely to be underdiagnosed. Certain occupations are more likely to be performed by men, and work-related exposure differences might contribute to the disproportional number of male patients given a diagnosis of leptospirosis. However, similar exposure risks during travel do not necessarily indicate similar rates of disease for persons of both sexes (*5**,**9*). In the Netherlands, risky vacation activities are found equally among men and women. However, these activities do not indicate an equal distribution of leptospirosis in men and women.

In addition, the disparity in the incidence of leptospirosis by sex of the patient was unlikely to be caused by differences in infecting serogroups because similar proportions of male and female patients were infected with serogroup Icterohaemorrhagiae. Therefore, we propose that sex of the patient might play a role in disease progression, which might influence the likelihood of diagnosis and reporting. Differences in health care–seeking behavior between male and female patients might also play a major role (*28*), However, genetic and physiologic differences that may affect disease manifestations in men should also be considered (*29**–**31*). Further research is needed to substantiate this hypothesis.

Our analysis has several major limitations. Use of passive surveillance data probably underestimates the total number of infections because mild cases of leptospirosis are less likely to be diagnosed. Furthermore, nationwide access to laboratory confirmation of leptospirosis has changed over time. In the early years of the study period, many features of the surveillance system were different, including diagnostic methods available, knowledge of existing serovars, collation of data, and clinician awareness of leptospirosis. However, the early data have contributed to our understanding of changes in leptospirosis over time because this dataset is comprehensive and includes an entire national cohort for >80 years.

We conclude that the effective surveillance system in the Netherlands, combined with adequate control measures, has reduced the incidence of leptospirosis in this country. Efforts to prevent imported infections should include providing better information on risks to travelers and greater awareness by clinicians about development of leptospirosis in persons with a history of travel abroad.
